# Outcomes of Treated Human Granulocytic Ehrlichiosis Cases

**DOI:** 10.3201/eid0804.010222

**Published:** 2002-04

**Authors:** Alan H. Ramsey, Edward A. Belongia, Craig M. Gale, Jeffrey P. Davis

**Affiliations:** * Centers for Disease Control and Prevention, Atlanta, Georgia, USA; † Wisconsin Division of Public Health, Madison, Wisconsin, USA; ‡ Marshfield Medical Research Foundation, Marshfield, Wisconsin, USA

**Keywords:** ehrlichiosis, granulocytes, tick-borne diseases, human, health status, treatment outcome

## Abstract

We conducted a case-control study in Wisconsin to determine whether some patients have long-term adverse health outcomes after antibiotic treatment for human granulocytic ehrlichiosis (HGE). A standardized health status questionnaire was administered to patients and controls matched by age group and sex. Consenting patients provided blood samples for serologic testing. Among the 85 previously treated patients, the median interval since onset of illness was 24 months. Compared with 102 controls, patients were more likely to report recurrent or continuous fevers, chills, fatigue, and sweats. Patients had lower health status scores than controls for bodily pain and health relative to 1 year earlier, but there was no significant difference in physical functioning, role limitations, general health, or vitality measures. The HGE antibody titer remained elevated in one patient; two had elevated aspartate aminotransferase levels. HGE may cause a postinfectious syndrome characterized by constitutional symptoms without functional disability or serologic evidence of persistent infection.

Human granulocytic ehrlichiosis (HGE) is a recently identified tickborne infectious disease caused by a bacterium species of the genus *Ehrlichia* that preferentially infects granular leukocytes (*1*). HGE was first described in the United States in 1994 in residents of Wisconsin and Minnesota (*2*). During 1986 through1997**,** 449 HGE cases were identified in the United States, mostly in the Northeast and Upper Midwest, despite limited reporting requirements (*3*). The primary vector of HGE is *Ixodes scapularis* (*4*), commonly known as the deer tick, which is also the vector of Lyme disease.

Onset of clinical signs and symptoms of acute HGE typically follow a 5- to 10-day incubation period. The acute illness is nonspecific and often includes fever, chills, headache, and myalgia. Abnormal laboratory findings may include leukopenia, thrombocytopenia, and mildly elevated liver enzymes (*5*). Seventeen percent to 56% of patients with HGE are hospitalized, and the case-fatality rate may be 0.7%-4.9% (*3*,*5*). Patients treated with doxycycline usually defervesce within 24 to 48 hours (*5*).

The acute phase of HGE is well characterized. The potential for persistent infection has been suggested but has not been evaluated. We conducted an exploratory case-control study of patients previously treated for HGE to assess health status, symptoms, and changes in serologic status.

## Methods

### Case Definition

The Centers for Disease Control and Prevention (CDC) HGE case definition was used. A confirmed case of HGE was defined as any acute febrile illness with laboratory confirmation consisting of 1) a fourfold or greater change in antibody titer to *Ehrlichia equi* by immunofluorescence antibody (IFA) test, 2) amplification of specific ehrlichial DNA sequences by polymerase chain reaction (PCR), or 3) demonstration of bacterial microcolonies (morulae) in leukocytes together with a single elevated titer**.** A probable case was defined as an acute febrile illness with a single elevated antibody titer >1:64 or presence of morulae (*6*).

### Patients

Both confirmed and probable cases of HGE were included in the case-control study. Although HGE officially became a notifiable disease in Wisconsin on April 1, 2000, we identified 111 cases of HGE in northwestern Wisconsin through specific surveillance activities from May 1996 to December 1998 (*7*). These included 86 (77%) confirmed and 25 (23%) probable cases. The 1996 cases were detected by laboratory-based surveillance within the Marshfield Clinic system, a network that provides health care to persons in northern and northwestern Wisconsin. The 1997 and 1998 cases were detected through active surveillance in a 13-county region in northwestern Wisconsin.

We selected 225 controls from a pool of approximately 880,000 living Wisconsin residents who had received medical care from the Marshfield Clinic regional network. Controls were randomly selected from the same zip codes as patients and frequency matched on age group (<19, 20-39, and 10-year intervals for persons >40 years old) and gender. A structured questionnaire on health status and symptoms was administered by telephone to patients and controls. The survey consisted of items from six domains of the Medical Outcomes Trust 36-Item Short Form Health Survey (SF-36) (*8*), including questions about physical function, role limitations due to physical health problems, bodily pain, general health, vitality (energy vs. fatigue), and health relative to 1 year earlier. In each domain, a higher score indicated a better health state. In addition, participants were asked if they had experienced the following symptoms continuously or repeatedly in the previous year: fever, fatigue, shaking chills, sweats, muscle aches, headache, joint pains, muscle weakness, nausea, cough, shortness of breath, diarrhea, vomiting, poor appetite, or confusion. The choice of signs and symptoms was based on known clinical manifestations of acute HGE (*2*,*5*,*7*).

Appropriate informed consent was obtained, and clinical research was conducted in accordance with guidelines for human experimentation as specified by the U.S. Department of Health and Human Services and the Marshfield Medical Research Foundation.

### Diagnostic Methods

 Case-patients were asked to provide blood samples for HGE serologic testing and measurement of aspartate aminotransferase (AST). We performed polyvalent IFA on sera using *E. equi* substrate (ProtaTek International, St. Paul, MN) and fluorescein isothiocyanate-conjugated goat anti-human immunoglobulin (Kallestad Diagnostics, Chaska, MN) diluted to 1:100. *E. equi* is closely related or identical to the agent that causes HGE (*1*,*9*).

### Statistical Analysis

Data were analyzed by using univariate logistic regression models with calculation of odds ratios (OR) and 95% confidence intervals (CI) by using SAS software (Version 6.12, SAS Institute, Cary, NC); p values <0.05 were considered statistically significant, and all are two-sided.

## Results

We enrolled 85 (77%) of 111 persons with previously treated HGE (patients) and 102 (45%) of 225 controls. Of the 111 patients, we were unable to contact 13 (12%); 8 (7%) refused participation; 4 (4%) had since died of causes unrelated to HE; and 1 (1%) did not complete the survey. Among the 225 controls, we were unable to contact 56 (25%); 61 (27%) declined to participate; and 6 (3%) surveys were incomplete.

There were 73 (86%) confirmed and 12 (14%) probable cases of HGE. Among confirmed cases, 32 (44%) were confirmed by serology alone; 13 (18%) by serology and polymerase chain reaction (PCR); 10 (14%) by PCR alone; 9 (12%) by serology, PCR, and presence of intracytoplasmic morulae; 6 (8%) by serology and presence of morulae; and 3 (4%) by PCR and morulae. Of the 12 probable cases, 9 (75%) had a single IFA serologic titer ≥64, and 3 (25%) had intracytoplasmic morulae identified in blood. The median age of patients was 58 years (range 6-88 years), and the median age of controls was 57 years (range 6-88 years); 66% of patients and 73% of controls were male. Data on coexisting conditions were available for all 102 controls and 39 (53%) of 73 confirmed cases. Comparison of the 39 cases and 102 controls demonstrated no significant difference in the prevalence of cancer (p=0.36), stroke (p=0.49), heart disease (p=0.89), or diabetes (p=0.65).

For HGE patients, the median interval from illness onset to telephone interview was 24 months (range 10-40 months). Twelve patients had onset of HGE in 1996, 35 in 1997, and 38 in 1998. The illness was severe enough in 32 (38%) patients to require hospitalization. All 85 enrolled patients received some form of antibiotic treatment for acute illness. Of the 59 patients for whom we have sufficient information, 53 (90%) were prescribed doxycycline for >7 days. The median interval from illness onset to initiation of treatment was 7 days. Nineteen (22%) enrolled patients had evidence of Lyme disease during the acute illness, either the characteristic rash, erythema migrans (14 patients), or seroconversion to *Borrelia burgdorferi* (7 patients). Six (8%) of 76 enrolled patients tested also seroconverted to *Babesia microti* after the acute illness.

Compared with controls, patients were more likely to report the following constitutional symptoms either continuously or repeatedly during the previous year: fevers, chills, sweats, and fatigue (Table 1). Patients had lower SF-36 health status scores for bodily pain (p=0.03) and health compared with 1 year earlier (p=0.02), but no differences existed for physical function, role limitations due to physical health, general health, or vitality (Table 2). When probable cases were excluded from analysis, confirmed patients were also more likely to report fevers (OR 4.1, 95% CI 1.0-15.9), chills (OR 3.8, 95% CI 1.3-11.4), sweats (OR 2.8, 95% CI 1.4-5.8), and fatigue (OR 1.7, 95% CI 1.0-3.1) during the previous year. Confirmed cases also had lower SF-36 scores for relative health (p=0.02), but no significant differences existed for bodily pain, physical function, role limitations due to physical health, general health, or vitality. When asked about their health status relative to before infection, 39% of patients believed their current health was “somewhat worse” or “much worse.”

**Table 1:** Recurrent or continuous symptoms experienced during the preceding 12 months by human granulocytic ehrlichiosis patients and controls, Wisconsin**,** 1999

Symptom	Patients (N=85) number (%)	Controls (N=102) number (%)	OR	95% CI
Fevers	12 (14.1)	3 (2.9)	5.4	1.7-24.4
Shaking chills	16 (18.8)	5 (4.9)	4.5	1.7-14.3
Sweats	31 (36.5)	16 (15.7)	3.1	1.6-6.3
Fatigue	47 (55.3)	38 (37.3)	2.1	1.2-3.7
Confusion	16 (18.8)	12 (11.8)	1.7	0.8-4.0
Muscle weakness	27 (31.8)	30 (29.4)	1.1	0.6-2.1
Headache	21 (24.7)	13 (12.8)	1.1	0.7-1.9
Muscle aches	40 (47.1)	44 (43.1)	1.0	0.7-1.5
Joint pains	50 (58.8)	54 (52.9)	0.9	0.6-1.2
Nausea	14 (16.5)	15 (14.7)	0.8	0.5-1.4

OR = odds ratio; 95% CI = 95% confidence interval.

**Table 2:** Mean scores and standard errors (SE) in six domains of the Medical Outcomes Study 36-Item Short-Form Health Survey in patients and controls^a^

Domain	Patients mean (SE)	Controls mean (SE)	p
Physical function	77 (2.3)	74 (2.8)	.83
Physical health-related role limitations	68 (4.1)	74 (3.7)	.16
Bodily pain	62 (2.9)	69 (2.5)	.03
General health	59 (1.4)	59 (1.4)	.64
Vitality	57 (2.4)	58 (2.1)	.46
Relative health	45 (2.4)	52 (1.8)	.03

^a^Optimal score in each category is 100.

The presence of continuous or recurrent constitutional symptoms and the duration of acute illness were not correlated. Patients who were hospitalized or who started antibiotics more than 14 days after onset of illness were no more likely to experience recurrent symptoms than those who received antibiotic treatment within 14 days. Similarly, patients with 1) a preexisting chronic illness, 2) intragranulocytic morulae in an acute-phase blood smear, 3) laboratory evidence of concurrent Lyme disease or babesiosis, 4) anemia, or 5) a high acute- or convalescent-phase reciprocal HGE IFA antibody titer (>512) were no more likely than the other patients to experience one or more of the recurrent or continuous symptoms.

Serum specimens were submitted for serologic testing by 70 (82%) of 85 patients. The HGE IFA antibody titer remained elevated (>1:64) in one (1.4%) of 70 specimens tested. This patient had a very high titer after acute infection (1:2,048), which remained elevated (1:256) 1 year later. He experienced continuous or recurrent fatigue, vomiting, and headaches. Two (2.9%) of 69 patients had elevated (>100 U/L) serum AST levels; one complained of continuous or recurrent chills, sweats, and fatigue.

## Discussion

Our results demonstrate that some patients with treated HGE may experience more fevers, chills, sweats, and fatigue than controls 1-3 years after onset of illness. Some patients also experience more bodily pain and have a poorer perception of their health compared with 1 year ago than controls, but they do not have any functional disability. Except for bodily pain, these findings persisted when only confirmed cases were included in the analysis. We found no serologic evidence to suggest the occurrence of persistent ehrlichial infection. These symptoms may therefore be attributed to a postinfectious syndrome rather than persistent or recurrent infection.

Few previous studies have evaluated the long-term serologic profile of treated HGE. Results of one study of treated HGE demonstrated that antibody titers remained elevated 11 to 14 months after onset in 5 of 10 patients tested (*10*). In another study of HGE patients, not all of whom were treated, Bakken et al. detected *E. equi* antibodies in 11 (46%) of 24 patients at 12 months, 4 (44%) of 9 at 18 months, and 2 patients (denominator not reported) at 30 months (*5*). The same researchers found that sera from 71 (15%) of 475 asymptomatic residents of northwestern Wisconsin contained antibodies to *E. equi* (*11*). In our study, only 1 of 70 patients had a persistently elevated antibody titer; however, specimens were collected more than 1 year after acute onset in approximately 95% of patients, and the geographic distribution of patients differed from that of the Bakken study.

Animal studies and case reports have suggested the possibility of chronic or recurrent HGE. Experimental ehrlichiosis infections in dogs and horses have demonstrated the presence of *E. canis* and *E. equi*, respectively, in tissues up to 2 months after treatment with doxycycline (*12*,*13*). An HGE patient from Wisconsin was one of the first anecdotal reports to suggest this possibility in humans. In that case, serologic evidence of a chronic ehrlichial infection or reinfection was identified 3 years after treatment for Lyme disease and HGE coinfection (*14*). Another study from Connecticut included one patient who had a specimen positive by PCR 7 weeks after treatment, indicating a possible persistent ehrlichial infection. In that study, PCR was used to demonstrate the presence of ehrlichial DNA in the blood of some patients who were seronegative (*15*). Dumler and Bakken detected HGE agent DNA by PCR 2 to 30 days after illness onset in four patients; two of them had no HGE antibody detectable at the time (*16*). Compared with the serology testing used in this study, PCR may be a more sensitive assay in detecting late ehrlichial infection (*17*).

Our study used current national case definitions for HGE (*6*). These criteria allow for positive PCR alone as laboratory confirmation. Ten (14%) of the 73 confirmed HGE cases were confirmed by positive PCR alone. However, a recent report by the American Society of Microbiology’s Task Force on Consensus Approach for Ehrlichiosis (CAFÉ) considers positive PCR alone (without other laboratory support) to represent probable laboratory evidence of HGE (*18*). If we had applied CAFÉ criteria to our study, we would have had 63 (74%) confirmed and 22 (26%) probable cases of HGE.

The primary outcome measures in this study were based on self-reported symptoms. Without laboratory confirmation of persistent infection, recall bias should be considered as a possible explanation for the findings. Because of the severity of their past illness and because this was not a blinded study, HGE patients may have been more aware of their constitutional symptoms and had better recall of them than controls. Selection of controls is another potential source of bias if they were less likely to have coexisting chronic diseases compared with cases. However, we found no statistical difference in the prevalence of cancer, stroke, heart disease, or diabetes between cases and controls.

Previous studies of various infectious diseases have suggested that convalescence from illness is significantly dependent on the emotional state of the patient. In those studies, as in ours, fatigue was often a persistent symptom (*19*–*21*). In a study of recovery from influenza, Imboden noted that “delayed recovery following acute self-limited illness occurs in persons who respond to psychological tests in patterns characteristic of depression-prone patients” (*19*). We did not collect baseline psychological data, but the subset of HGE patients with recurrent or persistent constitutional symptoms may have been psychologically predisposed to a protracted convalescence.

In summary, we found that a subset of patients with HGE have persistent constitutional symptoms 1-3 years after treatment, without functional disability. Further research is needed to determine whether these symptoms are related to the previous ehrlichial infection or other causes. In the absence of serologic evidence of persistent infection, we believe these symptoms are most likely due to a postinfectious syndrome. Further studies that use PCR testing of convalescent-phase samples would be helpful to exclude the remote possibility of persistent *Ehrlichia* infection.
